# Measuring and Explaining Socioeconomic Inequalities in Public Healthcare Utilization in Western Iran: Evidence from a Cross-sectional Survey 

**Published:** 2018-05-14

**Authors:** Shahab Rezaeian, Mohammad Hajizadeh, Satar Rezaei, Sina Ahmadi, Ali Kazemi Karyani, Yahya Salimi

**Affiliations:** ^1^Research Center for Environmental Determinants of Health, Kermanshah University of Medical Sciences, Kermanshah, Iran; ^2^School of Health Administration, Faculty of Health, Dalhousie University, Halifax, Canada; ^3^Social Development and Health Promotion Research Center, Kermanshah University of Medical Sciences, Kermanshah, Iran

**Keywords:** Socioeconomic status, Inequalities, Concentration index, Horizontal inequity index, Healthcare, Iran

## Abstract

**Background:** Equity in healthcare utilization is a major health policy goal in all healthcare systems. This study aimed to examine socioeconomic inequalities in public healthcare utilization in Kermanshah City, western Iran.

**Study design:** A cross-sectional study.

**Methods:** Using convenience sampling method, 2040 adult aged 18-65 yr were enrolled from Kermanshah City in 2017. A self-administrated questionnaire was used to collect data on sociodemographic characteristics, socioeconomic status, behavioral factors, and utilization of public healthcare services (inpatient and outpatient care) over the period between from May to Aug 2017. The concentration index (C) was used to measure and decompose socioeconomic inequalities in the utilization inpatient and outpatient care in public sector. The indirect standardization method was used to estimate the horizontal inequity (HI) indices in inpatient and outpatient care use.

**Results:** The utilization outpatient (C=-0.121, 95% CI: -0.171, -0.071) and inpatient care in public sector (C=-0.165, 95% CI: -0.229, -0.101) were concentrated among the poor in Kermanshah, Iran. Socioeconomic status, health-related quality of life, marital status and having a chronic health condition were the main determinants of socioeconomic-related inequalities in the utilization of inpatient and outpatient care in public sector among adults. The distributions of outpatient (HI=-0.045, CI: -0.093 to 0.003) and inpatient care (HI= -0.044 95% CI: -0.102, 0.014) in Kermanshah were pro-poor. These results were not statistically significant (*P*<0.05).

**Conclusions:** The utilization of public healthcare services in Iran are pro-poor. The pro-poor distribution of inpatient and outpatient care in public facilities calls for initiatives to increase the allocation of resources to public facilities in Iran that may greatly benefit the health outcomes of the poor.

## Introduction

Equity in health and healthcare is one of the main policy objectives in many healthcare systems^1,2^. Although equity has been used to measure the performance of healthcare systems ^3,4^, there is some uncertainty over the definition of equity in healthcare^5^. Nonetheless, there is broad agreement among policymakers and the general public on the principle that healthcare services should be financed according to the ability to pay and utilized primarily according to need^6^.

Equity in healthcare utilization is considered an important policy objective in Iran and Iranian Government has undertaken several reforms to achieve greater equity in the healthcare sector. The modern healthcare reforms in Iran began with the introduction of the Public Medical Service Insurance Coverage Act (PMSICA) in 1995 that extended health insurance coverage to the rural residents previously uninsured. Other initiatives such as Urban Inpatient Insurance Scheme (UIIS), the Rural Health Insurance Scheme (RHIS) were also implemented to improve the equity in healthcare services in Iran ^7^. The Health Sector Evolution Plan (HSEP) was also implemented in May 2014 to ensure equal access to services provided in all public hospitals^8^.

Several studies examined equity in healthcare use in different countries^9-12^. These studies reported substantial inequity (unfair inequality) in the utilization of healthcare across different social groups. The current literature highlighted inequalities in healthcare utilization among different socioeconomic status (SES), age, gender, employment status groups as well as across different regions. For example, SES was indicated as one of the main factors affecting access and utilization of healthcare in China ^13^. A pro-rich distribution was found in the utilization of public and private healthcare in Nepal ^12^. In Iran hat higher SES groups had less unmet need for ambulatory healthcare than their poorer counterparts ^14^.

Although several studies assessed the effect of SES on the utilization of healthcare services in different countries^9,15-17^, there is scant literature^10,18^ examined socioeconomic-related inequalities in healthcare utilization using summary measures of inequalities such as the concentration () index and horizontal inequity index (). Thus, using a cross-sectional study conducted in 2017 in Kermanshah City, western Iran, we aimed to measure and explain socioeconomic inequalities in the utilization of healthcare services.

## Methods

### Study setting

The current study was carried out in the capital of Kermanshah Province, Kermanshah City. This province located in the west of Iran and consists of 14 counties. It is bordered by Kurdistan Province to the north, Ilam Province to the south and Hamadan and Lorestan provinces to the east. This is bordered with Iraq to the west. The province had a total population of roughly two million in 2016, of which one million reside in Kermanshah City.

### Study population, sample size, and sampling method

A cross-sectional study was carried out from May to Aug 2017 to obtain data on the utilization of outpatient and inpatient care in the public sector among 2040 adults 18 yr and above in Kermanshah. The samples were chosen in two stages. Firstly, the city was divided into five areas of northern, southern, western, eastern and central. Then, convenience sampling method was used to select the equal samples from each area.

### Data collection and variables

A self-constructed questionnaire was used to collect data. The questionnaire was divided into two parts. The first part included questions about age, sex, health insurance coverage, educational attainment level, marital status, household durable assets (having car, house ownership and its square meter, room per capita, dishwasher, TV, vacuum cleaner, personal computer or laptop, access to Internet, cell phone, motorcycle, hood, bathroom and type of kitchen) of the study population. The second part of the questionnaire collected information on the utilization of inpatient and outpatient care, chronic health conditions. This part also included EuroQol 5-dimensions -3-level (EQ-5D-3L) questionnaire to measure health-related quality of life (HRQoL) using Iranian value set of EQ-5D-3L health states.

The main outcome variables in this study were two binary variables of utilization of outpatient and inpatient care provided by public providers. The outpatient care utilization was measured using the following question “Have you utilized any outpatient care (e.g., doctor visits, emergency care, medical tests, and routine exams) provided in public sector during the last 2 months?” The utilization of inpatient care was measured using the following question “Have you used inpatient care (e.g., stay in hospital more than 24 h) provided in public hospitals in the last 12 months?” We used age, sex, the presence of a chronic disease and HRQoL score as need factors and marital status, health insurance coverage, and socioeconomic status (SES) as non-need factors in our analysis^10-12,19^.Principal component analysis (PCA) techniques ^20-22^ were used to construct an SES indicator of participants. The SES indicator was measured using the durable properties of households (e.g., household durable assets such as owning, number of room per capita, type of house ownership, house square meter, hood, bathroom, car, computer/laptop, access to internet, freezer, dishwasher, TV and type of cooling and heating system) and individual’s level of education to measure socioeconomic status of household. The suitability of these variables in the PCA was checked using Bartlett's Test of Sphericity (BTS) and the Kaiser-Mayer-Olkin (KMO) index. The KMO index was 0.79 and BTS was also statistically significant (χ2 = 4500.70, *P* <0.001,) indicating the suitability of including these variables in the PCA. The SES indicator of participants was used to divided individuals into five SES quintile groups (1=poorest and 5=richest). To obtain the HRQoL score of the participants, we used the Iranian value set for EQ-5D-3L health states. This value set extracted by visual analogue score (VAS). More details about this model can be found elsewhere ^23^. The HRQoL varies between 0 and 1.

### Statistical Analysis

First, we measured and decompose socioeconomic inequalities in the utilization of outpatient and inpatient care in public sector. Then, we measured horizontal inequities in the utilization of outpatient and inpatient care in public sector.

*Measuring and decomposing inequalities in outpatient and inpatient care* :We used the C index to measure socioeconomic-related inequality in the utilization of both outpatient care and inpatient care among adults in Kermanshah City. The following formula was used to calculate the concentration index ^24:^

(1) $$\text{C}=\frac{2\text{*cov}\left({\text{y}}_{\text{i}}{\text{ r}}_{\text{i}}\right)\;}{\text{μ}},$$

where indicates the mean of the health variable interest (i.e., the use of inpatient or outpatient care) for the total sample, shows the outcome variable for individual , is the fractional rank in the SES distribution for the individual. The index ranges between -1 and +1. If the sign of the index is positive (negative), the use of inpatient/outpatient care is more concentrated among high (low) SES individuals^25^. We applied the Wagstaff’s correction^26^ to normalize the s (i.e. multiplying s by ), because the outcome variables of interest in this study are binary.

The can be decomposed to identify the contribution of each factors to the socioeconomic-related inequality in utilization of inpatient/outpatient care. If we have the following linear regression model linking our outcome variable, , to a set of predictor’s factors:

(2) $$y=\alpha +\underset{k}{\sum }\beta_{k}\;x_{k}+\;\varepsilon$$

Based on the association between and, the C index for dependent variables can be decomposed as follows^27^:

(3) $$C=\underset{k}{\sum }\left(\frac{\beta_{k}{\bar{x}}_{k}}{\mu }\right)C_{k}+\frac{GC_{\varepsilon }}{\mu },\;$$

where denotes the C index for the dependent variable, the ,C_k_, is the concentration index for x_k_. The $\frac{\beta_{k}{\bar{x}}_{k}}{\mu }$ is the elasticity of the outcome variable, y, with regard to the explaining variable and the last term,$\frac{GC_{\varepsilon }}{\mu }$ is a residuals component. The decomposition of the normalized C can be written as:

(4) $$C_{normalized}=\frac{C}{1-\mu }=\frac{\sum_{k}\left(\frac{\beta_{k}{\bar{x}}_{k}}{\mu }\right)C_{k}}{1-\mu }+\frac{\frac{GC_{\varepsilon }}{\mu }}{1-\mu }\;\;$$

Since the outcome variables in the study are binary variables, we used marginal effects obtained from the Probit model in the decomposition analysis^25^.

*Measurement of horizontal inequities in outpatient and inpatient care* : The Horizontal Inequity index (HI) was used to measure inequity in the utilization of inpatient care and outpatient care in public sector. The standardized indirect healthcare utilization was used to measure HI. The indirect standardized values of medical care can be calculated simply by running a regression of y, to a set of h healthcare need and z non-need predictor’s factors for the whole sample as follow^28^:

(5) $$y_{i}=G(\alpha +\;\sum_{j}\beta_{j}h_{ji}+\;\sum_{k}\gamma_{k}z_{ki})+\;\varepsilon_{i},\;\;$$

where y_i_ denotes the outcome variable for individual i (e.g., utilization of inpatient care or outpatient care), h_j_ is a set of j need variables that we want to standardize and z_k_ is a set k of non-need variables that we do not want to standardize but we want to estimate the partial correlation with need factors. takes special form for the Probit model. Then, the predicted value for healthcare utilization was calculated for each individual using the following formula:

(6) $${\hat{y}}_{i}^{x}=\;G(\hat{\alpha }+\;\sum_{j}{\hat{\beta }}_{j}h_{ji}+\;\sum_{k}{\hat{\gamma }}_{k}{\bar{z}}_{ki})+\;\varepsilon_{i},\;\;$$

where ${\hat{y}}_{i}^{x}$ indicates the predicted value of healthcare utilization and ${\bar{z}}_{ki}$ indicates the mean of non-need variable k. The concentration index of the ${\hat{y}}_{i}^{x}$ indicates the C index for need-predicted utilization, $C_{need\_predicted}$. We applied the Wagstaff’s correction^26^ in the calculation of the $C_{need\_predicted}$ and measured the as follows^28^:

(7) $$HI=C_{normalized}-C_{need\_predicted}$$

The ranges were between -2 and +2. The positive (negative) sign of the index indicates pro-rich (poor) inequity, indicating the higher concentration of healthcare utilization among the rich (poor) after adjusting for healthcare need of individuals. The *P* -value less than 0.05 was considered to be significant and all data analysis was performed using Stata version 14.2 (StataCorp, College Station, TX).

## Results

Overall, 2040 adults aged 18-65 yr were enrolled of whom 61.1% (1247) were male. Sixty percent of the study participants were married and 80% had health insurance coverage. The average HRQoL score for all of the samples were 0.748 (Standard Deviation= 0.17). Thirteen-one percent of the study population had at least one chronic health condition. The descriptive characteristics of the participants by type of healthcare utilization are reported in Table 1. In addition, mean of HRQoL scores for the study population was 0.748 ± 0.17.

**Table 1:** Descriptive characteristics of study population by type of healthcare utilization, Kermanshah, Iran: 2017 (n= 2040)

**Variables**	**All individuals**		**Outpatient care**		**Inpatient care**	
	**n=2040**	**%**	**n=973**	**%**	**n=382**	**%**
**Need factors**						
Age groups (yr)						
18-30	779	38.2	333	42.7	103	13.2
31-45	765	37.5	326	42.6	103	13.5
≥46	496	24.3	314	63.3	176	35.5
Sex						
Male	1247	61.1	570	45.7	222	17.8
Female	793	38.9	403	50.8	160	20.2
Chronic health condition					
Yes	267	13.1	189	70.8	121	45.3
No	1773	86.9	44.2	2.5	261	14.7
**Non-need factors**						
Health insurance coverage					
Yes	1622	79.5	792	48.8	277	17.1
No	418	20.5	181	43.3	105	25.1
Marital status						
Never-married	717	35.1	285	39.7	94	13.1
Currently married	1215	59.6	612	50.4	243	20.0
Divorced/separated/ widowed	108	5.3	76	70.4	45	41.7
Socioeconomic status					
1st quintile	408	20.0	220	53.9	115	28.2
2nd quintile	409	20.0	217	53.1	84	20.5
3rd quintile	407	20.0	188	46.2	54	13.3
4th quintile	408	20.0	179	43.9	63	15.4
5th quintile	408	20.0	169	41.4	66	16.2

The prevalence of utilization of outpatient services in the past two months was 47.7% (95% confidence interval [CI): 45.5% to 49.9%). The prevalence of inpatient services use in last year months was 18.7% (95% CI: 17.1% to 20.5%). The utilization outpatient care (=-0.121, 95% confidence interval [CI]: -0.171 to -0.071) and inpatient care (=-0.165, 95% CI: -0.229 to -0.101) in public sector were concentrated among the poor in Kermanshah, Iran.

The detailed contribution of need and non-need factors on socioeconomic-related inequalities in the probability of utilization of outpatient and inpatient care is presented in Table 2 and 3, respectively. Older age had a higher probability of utilization outpatient care in public sector (see the positive sign of marginal effects for older ages) (Table 2). Presence of a chronic health condition was also associated with 8.9 percentage point higher probability of utilization of outpatient care. There was an inverse association between higher HRQoL score and probability of outpatient care utilization. The results of the index for explanatory variables factors suggested that being men, single, having health insurance coverage and higher HRQoL were more concentrated among the people with high SES, whereas characteristics such as older age and having a chronic health condition are more prevalent among lower SES groups. The decomposition analysis indicated that the largest contribution to the observed inequality in utilization of outpatient care was SES (44.4%) and HRQoL score (40.7%). Besides these factors, marital status and having a chronic health condition also explained socioeconomic-related inequality in outpatient care utilization among adults in Kermanshah City.

**Table 2:** The decomposition analysis of inequality in the utilization of outpatient care in public sector in Kermanshah, Iran

**Variables**	**Marginal** **effects**	**Mean (**	**Elasticity**	**Concentration** **Index (**	**Contribution**		
					**Absolute**	**%**	**Summed %**
**Need Factors**							
Age groups (yr)							
18-30	Ref.	0.382	Ref.	Ref.	Ref.	Ref.	
31-45	-0.034	0.375	-0.026	-0.022	0.001	-0.9	
≥46	0.077	0.243	0.039	-0.053	-0.004	3.3	2.4
Sex							
Female	Ref.	0.611	Ref.	Ref.	Ref.	Ref.	
Male	0.045	0.389	0.037	0.008	0.001	-0.5	-0.5
Chronic health condition							
No	Ref.	0.869	Ref.	Ref.	Ref.	Ref.	
Yes	-0.089	0.131	-0.024	-0.142	0.007	-5.5	-5.5
HRQoL mean scores	-0.746	0.748	-1.169	0.022	-0.049	40.7	40.7
**Non-need Factors**							
Health insurance coverage							
No	Ref.	0.205	Ref.	Ref.	Ref.	Ref.	
Yes	-0.118	0.795	-0.197	0.048	-0.018	14.9	14.9
Marital status							
Never-married	Ref.	0.352	Ref.	Ref.	Ref.	Ref.	
Currently married	0.023	0.595	0.028	-0.054	-0.003	2.4	
Divorced/separated/widowed	0.153	0.053	0.017	-0.143	-0.005	3.8	6.2
Socioeconomic status							
1st quintile	Ref.	0.2	Ref.	Ref.	Ref.	Ref.	
2nd quintile	0.053	0.2	0.022	-0.399	-0.017	14.1	
3rd quintile	0.003	0.2	0.001	0.001	0.000	-0.0	
4th quintile	-0.032	0.2	-0.013	0.400	-0.010	8.5	
5th quintile	-0.041	0.2	-0.017	0.800	-0.026	21.6	44.2

**Note:**The absolute and percentage of contribution of the residuals was 0.003 and -2.261, respectively

Having health insurance coverage was associated with higher probability of utilization of inpatient care in public sector (see the positive sign of partial effect) (Table 3). The results suggested a positive association between having a chronic health condition and being women and probability of inpatient care utilization. Based on the decomposition results, the largest factors contributing to socioeconomic inequality in the utilization of inpatient care were HRQoL scores (26.8 %) and SES (14.1%) and chronic health condition (6.7%), respectively. 31.29% of socioeconomic-related inequality in utilization of inpatient care were explained by the explanatory variables included in the model.

**Table 3:** The decomposition analysis of inequality in the utilization of inpatient care in public sector in Kermanshah, Iran

**Variables**	**Marginal effects**	**Mean (**	**Elasticity**	**Concentration** **Index (**	**Contribution**		
					**Absolute**	**%**	**Summed %**
**Need factors**							
Age groups (yr)							
18-30	Ref.	0.382	Ref.	Ref.	Ref.	Ref.	
31-45	-0.007	0.375	-0.014	-0.022	0.001	-0.2	
≥46	0.092	0.243	0.119	-0.053	-0.008	4.7	4.5
Sex							
Female	Ref.	0.611	Ref.	Ref.	Ref.	Ref.	
Male	0.017	0.389	0.035	0.008	0.001	-0.2	-0.2
Chronic health condition							
Yes	-0.090	0.131	-0.063	-0.142	0.011	-6.7	-6.7
No	Ref.	0.869	Ref.	Ref.	Ref.	Ref.	
HRQoL mean scores	-0.410	0.748	-1.637	0.022	-0.044	26.8	26.8
**Non-need Factors**							
Health insurance coverage							
Yes	0.037	0.795	0.159	0.048	0.009	-5.7	-5.7
No	Ref.	0.205	Ref.	Ref.	Ref.	Ref.	
Marital status							
Never-married	Ref.	0351	Ref.	Ref.	Ref.	Ref.	
Currently married	-0.019	0.595	-0.061	-0.054	0.004	-2.4	
Divorced/separated/ widowed	0.031	0.053	0.009	-0.143	-0.002	0.9	-1.5
Socioeconomic status							
1st quintile	Ref.	0.2	Ref.	Ref.	Ref.	Ref.	
2nd quintile	-0.010	0.2	-0.011	-0.399	0.005	-3.3	
3rd quintile	-0.063	0.2	-0.067	0.001	0.001	0.0	
4th quintile	-0.036	0.2	-0.038	0.400	-0.019	11.4	
5th quintile	-0.009	0.2	-0.010	0.800	-0.010	5.9	14.1

**Note**: The absolute and percentage of contribution of the residuals was -0.114 and 68.7, respectively

The results of the index for inpatient and outpatient care are presented in Table 4. The distributions of outpatient (=-0.045, CI: -0.093, 0.003) and inpatient care (= -0.044 CI: -0.102, 0.014) in Kermanshah were pro-poor, these results, however, were not statistically significant at the 5% signiﬁcance level.

**Table 4:** Horizontal inequity indices for utilization of inpatient and outpatient care in public sector in Kermanshah, Iran

**Variables**	**Value**	**SE**	**95% CI**	***P*** **value**
Outpatient care				
C_normalized_	-0.121	0.025	-0.171, -0.071	0.001
C_need predicted_	-0.076	0.008	-0.090, -0.061	0.001
Horizontal inequity	-0.045	0.024	-0.093, 0.003	0.064
Inpatient care				
C_normalized_	-0.165	0.033	-0.229, -0.101	0.001
C_need predicted_	-0.122	0.012	-0.146, -0.098	0.001
Horizontal inequity	-0.044	0.030	-0.102, 0.014	0.140

## Discussion

We examined the socioeconomic inequalities in the utilization of outpatient and inpatient health services among adults in public sector in Kermanshah City. The overall prevalence of utilization of the inpatient care was 18.7% in the last year and 47.7% for the utilization of outpatient services in the last two months. The prevalence of public and private health services utilization was 60.8% and 53.8% in the last 12 months, respectively^29^. In Iran, 69.5% of adults aged 15 yr and older sought outpatient care in the past two weeks^30^.

Our study indicated that the concentration index for outpatient care and inpatient care was -0.109 and -0.153, respectively; suggesting the higher utilization of outpatient and inpatient care in public sector among the poor. This can be due to greater healthcare need of low SES individuals than their high SES counterparts. Socioeconomic inequality was investigated in outpatient services among 1608 participants (18 yr old and above) in Shiraz, Iran in 2012^10^. Consistent with our finding, they reported a higher prevalence of actual outpatient service among the poor. Studies in other countries have reported the higher concentration of healthcare services in both developed in developing countries. For example, a study in Afghanistan showed a higher utilization of public healthcare services among the poor ^31^. In the USA, the C was reported for the utilization of ambulatory care as -0.037^32^. In Brazil, one-year hospitalization rate was higher among higher SES individual over the period between 1998 and 2008 ^33^.

The results of decomposition analysis suggested that SES was one of the main factors explaining the concentration of the utilization of outpatient care in public sector among the poor in Kermanshah city. The lower HRQoL score also contributed to the higher prevalence of outpatient services in Kermanshah city. The decomposition results of inpatient care also revealed lower HRQoL score and SES as the main contributors of observed socioeconomic inequalities in the utilization of inpatient care. The contribution of SES to the concentration of outpatient and inpatient services among the poor in Iran may be explained by the greater accessibility of these services in public sector for the lower SES groups. Similar to our study, some previous studies^10^ indicated SES of individuals as one of the main factors affecting inequality in healthcare utilization. Socioeconomic inequality was investigated in healthcare utilization among the middle-aged and elderly in China in 2017 and found that the living standard (i.e. SES) of the study participants was the main factor contributing to socioeconomic inequalities in outpatient and inpatient visits ^19^.

Our findings suggest pro-poor horizontal inequities in the utilization of inpatient and outpatient service in Kermanshah City. The pro-poor horizontal inequities, however, were statistically weak ( for outpatient care=-0.045, *P* -value=0.064 and for inpatient care= -0.044; *P* =0.140). The pro-poor inequities in outpatient and inpatient care suggest that the poor compared to the rich in Kermanshah were more likely to use the outpatient and inpatient care in public sector after adjusting for the healthcare need. We did not observe significant differences in the extent of pro-poor inequities in the utilization of inpatient and outpatient services in Kermanshah City. The pro-poor distribution of public healthcare utilization also reported in the previous studies in other countries,^35^. Public healthcare services provided in health post and health clinic were pro-poor in Zambia^35^. Turkish Health Survey (THS) and measured HI index were used for different types of healthcare services^17^. Similar to our results, the latter study suggested pro-poor inequities in emergency care, inpatient care (and general practitioner care. Studies in Shina and Indonesia^36^ also indicated pro-poor horizontal inequity in outpatient care^15^. Another study in New Zealand also suggested pro-poor horizontal inequities in the utilization of general practitioner visits, outpatient visits, and inpatient care^37^.

The pro-poor horizontal inequities in healthcare use in Kermanshah, Iran, may be explained by the fact that we examined horizontal inequities in public healthcare services. It is expected to observe the higher rate of public healthcare use among the poor because public healthcare providers in Iran mainly deliver healthcare services for the low-income and uninsured. This is specially the case after the implementation of, a the HSEP reform in Apr 2014 that aimed to improve accessibility of healthcare, reducing the out of pocket for inpatient care for patients admitted to public hospitals (6% of total healthcare expenditures for urban and 3% for rural residents and small towns with populations <20 000)^38^. There is an evidence of an increase in the hospitalization rate after the implementation of the HSEP reform^39^.

Our study subjected to some limitations and the results of the study should be interpreted in light of these limitations. Firstly, data on the utilization of outpatient care last 2 months and inpatient care in the last 12 months are self-reported, which are subject to recall bias^40^. Second, the design of the study was cross-sectional; thus, the caution is needed in interpreting causal relations. Third, the residual contributed significantly to socioeconomic inequality in inpatient services. There are other factors that have an impact on the socioeconomic distribution of inpatient care not included in our model. Finally, the use of convenience sampling method to select study participants limits the generalizability of the study findings.

## Conclusions

Improving socioeconomic inequalities in healthcare utilization is an important policy objective in all countries. Our results indicate a pro-poor distribution of public healthcare services in Kermanshah, Iran. The concentration of public healthcare services among the low SES groups in Iran has valuable health policy implications for improving the provision of healthcare for the low SES groups. The pro-poor utilization of public healthcare in Iran can be used for supporting greater resource allocation to public healthcare facilities which we found mainly provide health services to socioeconomically disadvantaged groups. The pro-poor distribution of public healthcare also calls for other initiatives to improve service quality in the public healthcare facilities in Iran. This, ultimately, lead to the improvement of the health outcomes among the poor.

## Acknowledgments

The authors gratefully acknowledge the Research Council of Kermanshah University of Medical Sciences (KUMS).

## Conflict of interest statement

The authors have no conflicts of interest to declare for this study.

## Funding

The study was funded and supported by the Research Deputy of Kermanshah University of Medical Sciences.

## Highlights

- The utilization rates of outpatient and inpatient services in Kermanshah City were 47.7% and 18.7%, respectively.
- The utilization outpatient and inpatient care in public sector were concentrated among the poor in Kermanshah City.
- SES and HRQoL were the two main contributors to the observed socioeconomic inequality in the utilization of outpatient care.
- The largest factors contributing to socioeconomic inequality in the utilization of inpatient care were HRQoL scores, SES, and chronic health condition.
- Pro-poor horizontal inequities in the utilization of outpatient and inpatient care in Kermanshah City.
